# A Predefined-Time Control for the Laser Acquisition in Space Gravitational Wave Detection Mission

**DOI:** 10.3390/s22187021

**Published:** 2022-09-16

**Authors:** Jinxiu Zhang, Peiji Wang, Xiaobin Lian, Lang Lu, Wei Liu

**Affiliations:** 1 School of Aeronautics and Astronautics, Sun Yat-sen University, Shenzhen 518038, China; 2 School of Physics and Astronomy, Zhuhai Campus, Sun Yat-sen University, Zhuhai 519082, China; 3 Shanghai Institute of Satellite Engineering, Shanghai 201109, China; 4 Institute of Space Science and Applied Technology, Shenzhen Campus, Harbin Institute of Technology, Shenzhen 518055, China

**Keywords:** gravitational wave detection, two-way laser link, acquisition phase, scanning guidance law, predefined-time stability

## Abstract

The establishment of a laser link between satellites, i.e., the acquisition phase, is a key technology for space-based gravitational detection missions, and it becomes extremely complicated when the long distance between satellites, the inherent limits of the sensor accuracy, the narrow laser beam divergence and the complex space environment are considered. In this paper, we investigate the laser acquisition problem of a new type of satellite equipped with two two-degree-of-freedom telescopes. A predefined-time controller law for the acquisition phase is proposed. Finally, a numerical simulation was conducted to demonstrate the effectiveness of the proposed controller. The results showed that the new strategy has a higher efficiency and the control performance can meet the requirements of the gravitational detection mission.

## 1. Introduction

**Figure 1:**
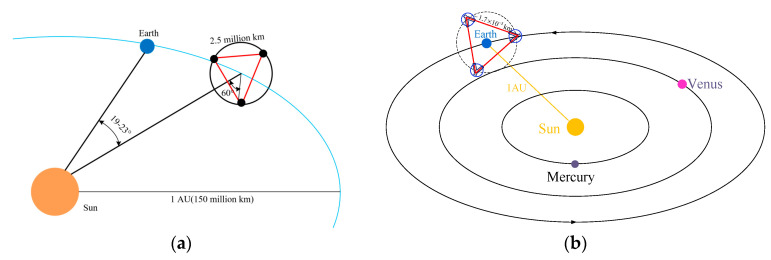
Orbital configuration of the LISA mission and the TianQin project. (**a**) LISA mission [5]. (**b**) TianQin project.

Gravitational waves (GWs) have been directly detected multiple times by the ground-based LIGO [1] and VIRGO [2] detectors since 2015 with the unremitting efforts of scientists [3]. However, the ground environment and limited arm length determine that ground-based detectors can only detect GWs in high frequency bands of about 10~1000 Hz. However, more abundant GWs are concentrated in lower frequency bands of about 0.1 mHz~1 Hz. The space-based detectors are an excellent solution, which can take advantage of the ultra-quiet environment in space and have a longer arm length. LIGO’s success gives a significant boost to the development of space-based GW detectors. LISA is a relatively early proposal of the space-based GWs detector project which started in 1993 [4]. After nearly 30 years of development, LISA has become the most mature and complete space GW detection project. LISA consists of a cluster of three identical spacecraft, flying in a quasi-equilateral triangular formation with an edge length of 2.5 million km in a heliocentric orbit with a semi-major axis of 1 AU. The LISA constellation is trailing the Earth at a distance of approximately 60 million Km or at a Constellation–Sun Earth angle of around 20° behind the Earth [5], which is depicted in Figure 1a. Each of the three identical spacecraft carries a measurement system consisting of two steerable telescopes associated with laser interferometer measurement systems. TianQin is a detector of GWs in the millihertz frequencies and was proposed in 2014 by Luo Jun [6,7]. TianQin is a space-based detector consisting of three drag-free spacecraft orbiting the earth as an approximately equilateral triangle constellation. The GWs are detected by measuring the relative distance between free falling test masses inside each spacecraft through the laser interferometer between each two spacecrafts. The TinQin Project conception is depicted in Figure 1b.

We collected some main GW detection programs all over the world which are listed in Table 1.

From Table 1 we can see that these space GW detection programs consist of at least three satellites forming an approximate equilateral triangle constellation with very large arm lengths. That is to say, there are three two-way laser links which need to be established.

With the development of space technology, the establishment of inter-satellite laser links has become a research hotspot in the field of space laser communication, gravity measurement and space GW detection. There are many studies on the acquisition phase of inter-satellite laser links. Scheinfeild [17] proposed four acquisition modes for satellite laser communication, namely: Stare/Stare mode, Stare/Scan mode, Scan/Scan mode and Scan/Stare mode. They are distinguished by the relationship among the laser divergence angle (LDA), uncertainty cone angle (UCA) and field of view (FOV), see Table 2. Stare/Stare mode is based on the condition that the LDA and FOV all are bigger than the UCA so that the acquisition sensor will detect the signal immediately when the laser is on. In, this mode, it is easy to establish the laser link, but it requires large laser power and ca not be used for long distances, so it is rarely used in practice. The Stare/Scan mode is based on the condition that the LDA is smaller than UNA and the FOV is bigger than the UCA, so the laser beam of the master one needs to scan the uncertainty cone with the guidance law and the slave one needs to keep still and stare at the uncertainty cone to wait for the signal. This is the most commonly used mode in practice. The Scan/Stare mode is when the LDA is bigger than UCA and UCA is bigger than FOV, so the laser beam of the master one needs to keep still and the slave one scans the uncertainty cone to search the signal. This mode also needs large laser power and is rarely used in practice. The Scan/Scan mode is when the laser beam of the master one and the slave one all need to perform scans to search for each other. This mode will reduce the probability of acquisition and is rarely used in practice. Furthermore, the characteristics of scanning methods such as spiral scanning, raster scanning, Lissajous scanning and rose scanning have been compared and analyzed [18]. Mahrdt [19] conducted a detailed study for the acquisition phase of the GRACE Follow-on mission. Tolker-Nielsen [20] introduced the inter-satellite laser link experiment of the SLIEX mission and gave a detailed description of the acquisition scenario. Hyde [21] designed a control law for the acquisition process of the LISA mission and conducted a simulation to verify the pointing performance. The pointing error was less than 8 nrad/Hz^1/2^ in the frequency band 1~100 mHz. Maghami [22] proposed an approach for the acquisition control of LISA formation to build the six laser links one by one, and the simulation results indicated that the LISA acquisition requirements can be met in a timely manner. Cirillo [23,24] designed a control system for the acquisition phase of the LISA constellation, built the six laser links one by one with the spiral scanning guidance low and implemented a Kalman filter to reject the disturbance. The simulations demonstrated that an acquisition strategy with the proposed control method is feasible. Zhang [25] proposed an acquisition scheme based on the differential power sensing method for the gravity measurement mission: two satellites scanned uncertainty cones simultaneously and independently. The laser beams were power-modulated at different frequencies instead of the commonly used single-way scanning method. The numerical simulation and experiments achieved a success rate of more than 99% and the acquisition time was significantly reduced. Gao [26] proposed a high-speed, high-precision, fully automatic acquisition scheme for the Taiji Program. They developed an improved differential power sensing combined with the down-sampling algorithm and the match filter algorithm and achieved 1µrad resolution and 220 s scanning time. Most of the current studies about the acquisition have focused more on the field of laser communication and less on the field of the GW detection. In addition, the studies shown above indicate that current schemes for the acquisition phase of the GW detection build the laser link one by one for six links which means more time costs. To reduce the acquisition time, we proposed a new configuration of satellite for the acquisition phase.

In recent years, many space applications require stringent time response constraints. To satisfy the requirements for the time response constraints, the finite-time stability [27,28,29,30] was proposed. Nevertheless, the finite-time stability often depends on the initial conditions of the system; that is to say, the finite time varies with the initial conditions. To solve this problem, the fixed time stability [31,32,33] was proposed where the time can be guaranteed to be a constant under the change of initial conditions. However, it is hard to obtain the direct relationship between the tuning parameters and the fixed stabilization time. Furthermore, a new method called prescribed-time stability [34,35] control was proposed where the control gains are functions of the desired convergence time, whereas the convergence time is a conservative estimation of the upper bound of the stabilization time so that it is often lager than the true convergence time. To overcome these problems above, Sanchez-Torres [36] developed the predefined-time stability control where the settling times can be considered as an explicit parameter and the predefined time is the minimum upper bound of the fixed stabilization time. As a new control strategy and powerful control method for the applications with stringent time response constraints, the predefined-time stability has been successfully applied in many fields and has achieved good results. Munoz-Vazquez [37] proposed a predefined-time robust stabilization for the robotic manipulators, Wang [38] proposed a predefined-time attitude control for the rigid spacecraft and Wu [39] proposed a predefined-time attitude stabilization control for the receiver aircraft in aerial refueling. The establishment of the inter-satellite laser link also requires stringent time response constraints. When the master satellite performs the scanning with guidance law step-by-step, the telescope is expected to point a certain direction within a certain time so that the salve satellite has enough time to respond and the laser beam can be guaranteed to fully cover the uncertainty cone. To the best of our knowledge, the predefined-time stability control theory has not been applied in the acquisition phase for the establishment of the inter-satellite laser link.

In view of above results, we investigated the laser acquisition problem for space-based GWs detection programs. The main contributions in this paper are:

(1) We proposed a new type of satellite equipped with two two-degree-of-freedom-telescopes. This configuration allows two laser links to be established at the same time, which means that the acquisition time will be drastically reduced;
(2) We proposed a predefined-time controller for the acquisition phase. This control method can guarantee that the telescope will point to the desired direction within the predefined time, which will greatly improve the probability of acquisition.

The remaining parts of this paper are Section 2 introduces the acquisition scheme. Section 3 develops the dynamic model for the platform. Section 4 designs the predefined-time controller. Section 5 is the simulation and Section 6 is the conclusions.

## 2. Acquisition Scheme

### 2.1. Satellite Platform Description

**Figure 2:**
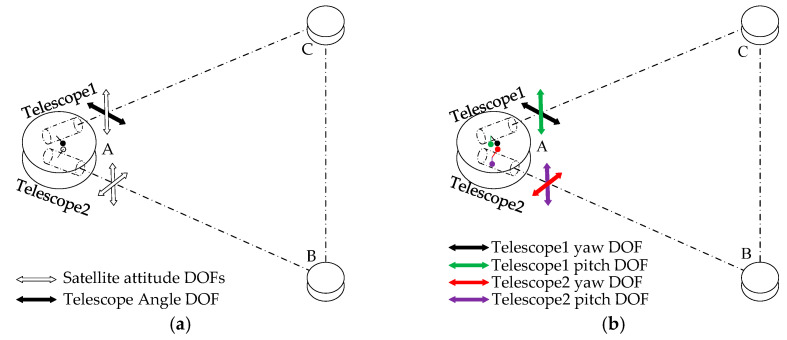
Control of the telescope pointing DOFs. (**a**) Three attitude DOFs and one telescope DOF; (**b**) Each telescope has two DOFs.

Almost all the current GWs detection programs have three satellites, and each has two telescopes, which forms an approximate equilateral triangle constellation with three laser interferometers. Obviously, there are three two-way laser links; namely, three arms need to be established. The two telescopes on one corner of the triangle, together with the corresponding telescope on the other two satellites, constitute two of three giant Michelson-Type interferometers through two-way laser links. The third arm is necessary to give independent information on the two polarizations of GWs for redundancy. These interferometers will detect low frequency GWs by measuring the distance change between the two released test masses which are in drag free mode. Because of the orbital perturbation, the constellation will no longer be a standard equilateral triangle configuration. That is to say, the angle between two telescopes will have a small angle change which will no longer be 60°. Therefore, the telescopes need to adjust their point direction to compensate for the angle change with a very high pointing accuracy. This also indicates that one satellite will track the other two satellites simultaneously which means each satellite needs at least four pointing degree-of-freedoms (DOFs). There are two alternatives which are depicted in Figure 2:

(1) By combining mechanisms controlling the one angle DOF between the telescope with the micro-thrusters controlling the three attitude DOFs of the entire satellite; this scheme is shown in Figure 2a;
(2) By two-axis mechanisms controlling each telescope with two DOFs; this scheme is shown in Figure 2b.

**Figure 3:**
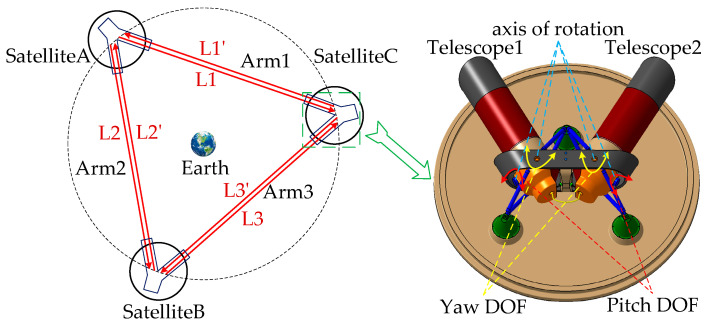
Configuration of the satellite platform for the GWs detection mission.

In this paper, we chose the second scheme which is described in detail in Figure 3. The telescope 1 (T1) of satellite A (SA) pointing to the satellite C (SC) is credited as L1, the telescope 2 (T2) of SA pointing to the satellite B (SB) is credited as L2, the T2 of SB pointing to SC is credited as L3 and the opposite directions are credited as L1′, L2′ and L3′, respectively. There are three two-way laser links in total: L1/L1′, L2/L2′ and L3/L3′.

### 2.2. Sensors and Actuators

Before the laser beam signal is detected, the absolute pointing accuracy of the telescope depends on the star trackers and telescope pointing angle sensors. The accuracy of the star trackers whose use has been suggested in the GWs detection mission is about 1 arcsec (3σ) and its field-of-view (FOV) is about 0.26 rad [24]. The read-out noise introduced by the telescope pointing angle sensor can be represented as white noise with a constant power spectral density equal to 10 nrad/Hz^1/2^ [24]. When the laser beam signal is detected, the acquisition sensor can be used as an attitude sensor to provide the relative attitude angle knowledge between two satellites. The acquisition sensor suggested in this paper is an array detector, which can replace the traditional scheme of combining Charged Coupled Device (CCD) and Quadrant Photodetector (QPD) to detect the weak laser signals from the sending satellite. The accuracy of the array detector we suggested is about 10 nrad/Hz^1/2^ [40]. Additionally, the obvious difference between the traditional satellite and the GWs detection satellite is that the GWs detection satellite has two cubic test mass for inertial reference. The test mass and its measurement system can be used as a sensor to measure the relative position and attitude between satellite and test mass with an extremely high accuracy. In this paper, it is considered as a sensor for high-precision attitude control in acquisition phase with an accuracy about 2 × 10^−9^ rad/Hz^1/2^ [41].

The actuators used in the acquisition phase include the micro-propulsion system and the telescope pointing actuator system. The micro-propulsion system is used to provide force and torque for the position and attitude control of the satellite. The thrust range of the micro-propulsion varies from 1 μN to 120 μN with a noise about 0.1 μN/Hz^1/2^ [42]. The telescope pointing actuator system is a two-axis mechanism which is used to provide force for the two-direction rotation control of the telescope. It will perform the scanning and compensate for the angle change between the telescope which is caused by the orbital perturbation. The telescope pointing actuation disturbance noise introduced by the actuator can be represented as white noise with a constant power spectral density equal to 60 nN/Hz^1/2^ [24].

### 2.3. Uncertainty Cone

In order to establish the laser links between the satellites, the laser beam signal from the sending satellite must be detected by the acquisition sensors in the receiving telescope. However, because of the long distance, navigation error, attitude accuracy and telescope pointing error, it is almost impossible to establish the links by initial pointing. Therefore, the attitude knowledge supplied by the attitude sensors must be relied upon. Therefore, the scanning process is necessary which means that the telescope on the sending satellite needs control of its pointing direction with a guidance law to cover the uncertainty cone of the receiving satellite until the laser beam signal is detected by the acquisition sensor in the opposite telescope. The acquisition phase is completed when all the telescopes receive the laser beam signal.

The uncertainty cone is the cone where the receiving spacecraft is expected to be located with respect to the sending spacecraft. The spacecraft attitude accuracy will be the relatively large contribution for the uncertainty cone which is limited by the accuracy of the star tracker. The navigation error is another factor but is associated with the orbit; earth orbit programs have a smaller navigation error than sun orbit programs. The other influencing factors such as calibration error, outgoing beam direction error and telescope pointing error are relatively small. These contributions will determine the size of the uncertainty cone which further affects the acquisition time.

### 2.4. Acquisition Procedure

- Scanning for Uncertainty Cone

**Figure 4:**
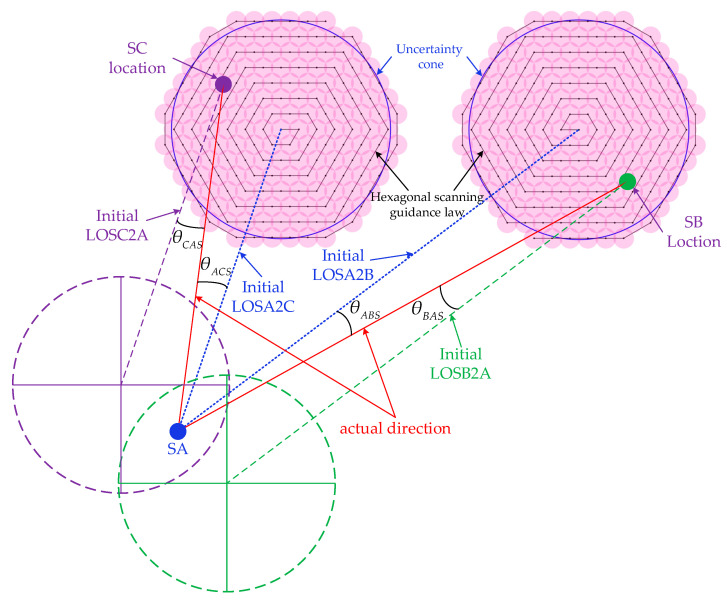
Schematic diagram of scanning for the uncertainty cone. Where initial LOSA2C and LOSA2B denote the initial line of sight of the T1 and T2 of SA towards SC and SB, respectively; initial LOSC2A and initial LOSB2A denote the initial line of sight of the T2 of SC and T1 of SB towards SA; *θ_ABS_* and *θ_ACS_* denote the angle offset between initial LOS of SA and actual direction toward SC and SB, respectively; *θ_CAS_* denotes the angle offset between the initial LOS of SC and the actual direction toward SA; *θ_BAS_* denotes the angle offset between the initial LOS of SB and the actual direction toward SA.

The first step of acquisition is to scan the uncertainty cone of the opposite satellite. This configuration we proposed above has a huge advantage for the acquisition phase which is that the two telescopes can scan their corresponding uncertainty cone at the same time. That means a much shorter acquisition time. SA will turn on its laser and use the ground-provided navigation data to control T1 to scan the uncertainty cone of SC meanwhile controlling T2 to scan the uncertainty cone of SB with the same hexagonal scanning guidance law but a different scan direction. The hexagonal scanning guidance law has been proved to be the most efficient discrete stepping scan method [19]. The purpose of the same law but a different direction is hopefully to reduce the disturbance of the satellite which is caused by the moving of telescope as much as possible. The detailed description is in Figure 4.

- The Establishment of Two-way Laser Links

**Figure 5:**
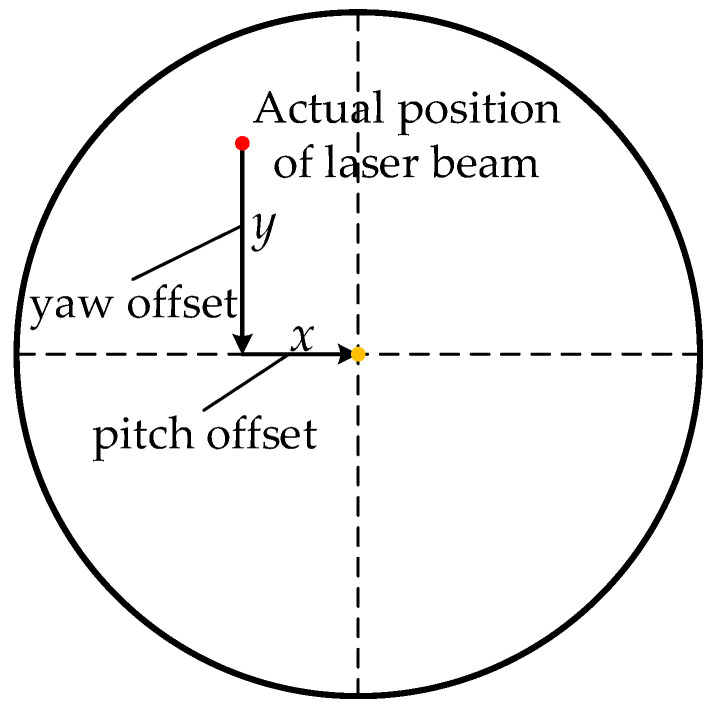
Signal detection on the array detector.

SA will perform scanning until the laser beam covers all the uncertainty cones of SB and SC. The SB and SC will keep their corresponding telescope staring at the uncertainty cone of SA at the same time and wait for the arrival of the laser beam from SA. Obviously, the FOV of the array detector must cover the whole uncertainty cone of SA to ensure that the laser signal from SA can be received. The laser beam will arrive the array detector at a certain time, then SB and SC will control their telescopes to point the direction of the laser beam according to the displacement information of the laser spot detected by the array detector and turn on their laser to illuminate SA. The laser spot on the array detector is shown in Figure 5. Because of the long distance between the three satellites, the laser beam will be very weak when arriving at the array detector of SA, so the signal will be drowned in noise. SA will turn off its laser when it finishes the scanning so that the laser signal from SB and SC can be detected. Then SA will control its two telescopes towards SB and SC, respectively, according to the direction of the incoming laser beam and turn on its laser. Finally, the two two-way laser links L1/L1′ and L2/L2′ will be established.

The *θ_ACS_* and *θ_ABS_* can be orthogonally decomposed to (*θ_ACp_*, *θ_ACy_*) and (*θ_ABp_*, *θ_ABy_*), respectively. The relationship between them can be is represented as:

(1) $$\left\{\begin{array}{c} \theta_{ABS}=\sqrt{\theta_{ABp}^{2}+\theta_{ABy}^{2}}\\ \theta_{ACS}=\sqrt{\theta_{ACp}^{2}+\theta_{ACy}^{2}} \end{array}\right.$$

Furthermore, according to Figure 5 we have:

(2) $$\left\{\begin{array}{c} \tan\theta_{ABp}=\frac{x}{f}\\ \tan\theta_{ABy}=\frac{y}{f} \end{array}\right.$$

where *f* denotes the equivalent focal length of the telescope. Similarly, the *θ_ABp_* and *θ_Aby_* can also be calculated.

- The Establishment of the Third Two-way Laser Links

**Figure 6:**
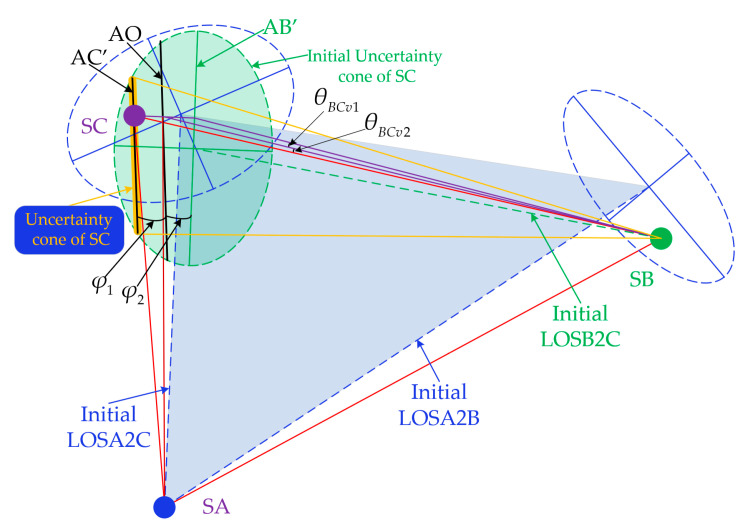
Schematic diagram of the third two-way laser link. Where the initial LOSB2C denotes the initial line of sight of the T2 of SB towards SC, segment AC’ denotes the projection of the actual direction between SA and SC on the circular plane area of the initial uncertainty cone of SC, while segments AO and AB’ denote the projection of the initial LOSA2C and the actual direction between SA and SC, respectively, *φ*_1_ denotes the angle between AC’ and AO, and *φ*_2_ denotes the angle between AO and AB’.

When the L1/L1′ and L2/L2′ are established, it is easier to establish the third one according to the high-accuracy attitude knowledge of the previous two links which is already available. As shown in Figure 6, according to Equations (1) and (2), we have:

(3) $$\left\{\begin{array}{c} \varphi_{1}\approx \theta_{ACp}\\ \varphi_{1}\approx \theta_{ABp} \end{array}\right.$$

The distance between the three satellites is almost equal, so we also have:

(4) $$\theta_{BCp}=\theta_{BCp1}+\theta_{BCp2}\approx \theta_{ACp}+\theta_{ABp}$$

So, the uncertainty cone of SC will be changed from a circular area to a narrow bar-like area according to the information of the actual direction of SB and SC. It will greatly reduce the establishment time of the third link. SB can use its T2 to scan the uncertainty cone of SC which is shown in Figure 6 as the orange bar-like area. When SC receives the laser signal from SB, it will control its T1 to point to SB and turn its laser. When SB finishes scanning and turns off its laser, it will find the laser signal from SC and control its T2 to point to SC then turn on its laser. Finally, the Constellation laser links are fully established; namely, the acquisition phase is completed.

## 3. Mathematical Model

### 3.1. Reference Coordinate Systems

Earth-Centered Inertial frame $\sum_{I}$(*Ex_i_y_i_z_i_*): the equatorial plane at epoch J2000 is used as a base plane, the origin is located at the mass center of earth, the *x_i_*-axis pointing to the mean vernal-equinox at epoch J2000, the *z_i_*-axis normal to the mean equator at epoch J2000 and the *y_i_*-axis completing a right-handed orthonormal reference system.

Orbital coordinate frame $\sum_{O}$(*Ox_o_y_o_z_o_*): the origin is located at the mass center of the satellite, the *z_o_*-axis is along the direction that from the satellite’s mass center to the Earth’s mass center, the *x_o_*-axis in the orbital plane is perpendicular to *z_o_*-axis and pointing to the motion direction and the *y_o_*-axis is perpendicular to the orbital plane and form a right-handed triad.

Spacecraft body coordinate frame $\sum_{0}$(*Ox_b_y_b_z_b_*): The origin of this coordinate system is located at the mass center of the satellite and its axes are fixed with the satellite body. The *z_b_*-axis is located on the angular bisector between the two telescopes, the *y_b_*-axis pointing toward the bottom panel and the *x_b_*-axis forming a right-handed triad.

Telescope swing mechanism coordinate frame $\sum_{1}$ and $\sum_{2}$(*Ox_1_y_1_z_1_* and *Ox_3_y_3_z_3_*): The origin is located at the telescope’s rotation center axis, and the *x_j_*-axis (*j* = 1,3) is along the telescope’s nominal line of sight, the *z_j_*-axis (*j* = 1,3) is along the axis of rotation and the *y_j_*-axis (*j* = 1,3) forms a right-handed triad. In addition, *j* = 1 for the left one and *j* = 3 for the right one.

Telescope body coordinate frame $\sum_{2}$ and $\sum_{4}$(*Ox_2_y_2_z_2_* and *Ox_4_y_4_z_4_*): This coordinate frame is fixedly connected on the telescope. The *x_j_*-axis (*j* = 2,4) is along the telescope’s line of sight, and the *z_j_*-axis (*j* = 2,4) is along the axis of rotation, then *y_j_*-axis (*j* = 2,4) can be determined by the right-handed rule. Additionally, *j* = 2 for the left one and *j* = 4 for the right one.

**Figure 7:**
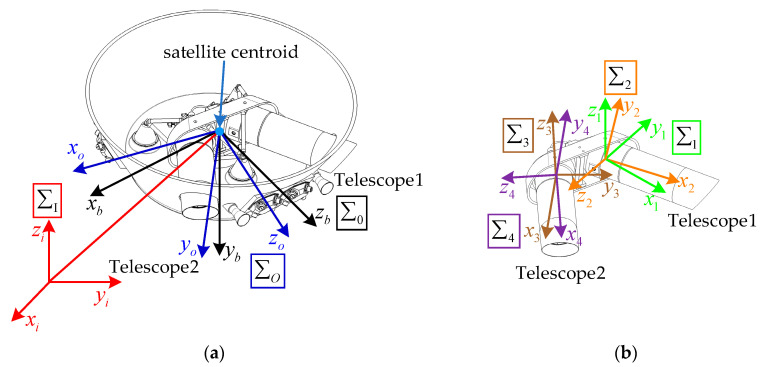
Reference coordinate systems. (**a**) Satellite coordinate systems; (**b**) telescope coordinate systems.

The detailed coordinate systems are shown in Figure 7.

### 3.2. Topological Relationship for the Satellite Platform

**Figure 8:**
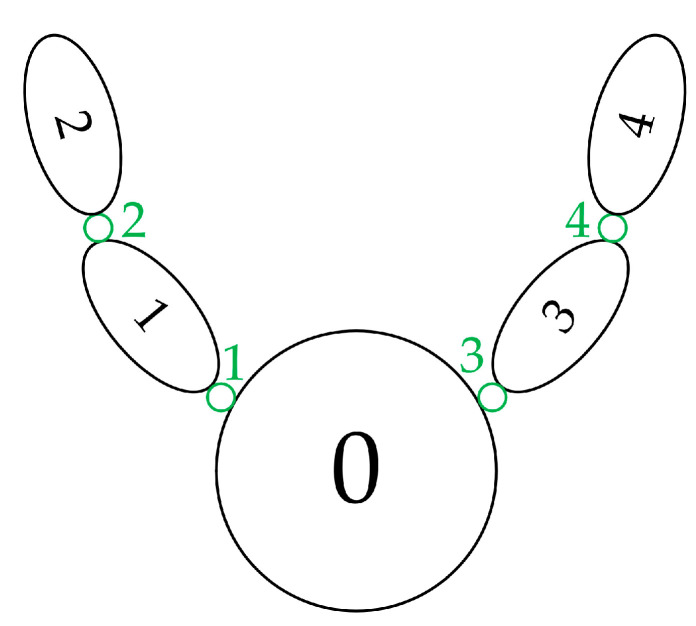
Schematic diagram of the topology of the satellite platform. The black numbers represent the body number and the green numbers represent the joint number.

**Figure 9:**
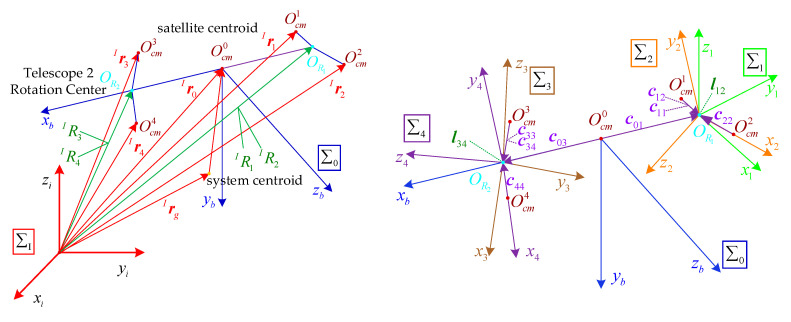
Vector relationship. $C_{ij}$ denotes the link vector for the centroid of body *i* body to joint *j*, $l_{ij}$ denotes the link vector from joint *i* to joint *j*, $l_{ij}=c_{ij}-c_{ii}$, $O_{cm}^{i}$ denotes the centroid of body *i*, $O_{R_{1}}$ denotes the rotation center of telescope 1, $O_{R_{2}}$ denotes the rotation center of telescope 2.

One satellite which carries two telescopes that each have two DOFs is a typical multibody system. We use the graph theory to describe the interconnection of these bodies mathematically. The topology of the satellite platform is shown in Figure 8. The satellite body is numbered 0, the telescope swing mechanisms are numbered 1 and 3 and the telescope bodies are numbered 2 and 4. These numbers correspond to the coordinate system numbers which is showed in Figure 9.

### 3.3. Mathematic Model for the Satellite Platform

According to the vector relationship, we can obtain the line velocity and angle velocity of the *i*-th body as:

(5) viIωiI=E3r0i×ITO3×3E3v0Iω0I+JTiJRiθ˙

where r0iI=riI−r0I, riI denotes the position vector of the centroid of body *i* in $\sum_{I}$, rgI denotes the position vector of the centroid of the spacecraft system in $\sum_{I}$, $\theta \in {\mathbb{R}}^{4\times 1}$ denotes the angle velocity of the four DOFs of two telescopes, $J_{Ti}$ and $J_{Ri}$ denote the Jacobian matrixes which can be expressed as:

(6) JT1=k1I×r1I−R1I,0,0,0JT2=k1I×r2I−R1I,k2I×r2I−R2I,0,0JT3=0,0,k3I×r3I−R3I,0JT4=0,0,k3I×r4I−R3I,k4I×r4I−R4I

where RiI denotes the position vector of the joint *i* in $\sum_{I}$:

(7) JR1=k1I,0,0,0JR2=k1I,k2I,0,0JR3=0,0,k3I,0JR4=0,0,k3I,k4I

So, we can obtain the kinetic energy of *i*-th body:

(8) Ti=12miviTIviI+12ωiITIiIωiI=12viIωiITmiE300IiviIωiI

and the whole kinetic energy of the satellite platform is:

(9) T=∑i=04Ti=12x˙bIθ˙THbHbtHbtTHtx˙bIθ˙

where $H_{b}\in {\mathbb{R}}^{6\times 6}$ denotes the inertia matrix of satellite body, $H_{t}\in {\mathbb{R}}^{4\times 4}$ denotes the inertia matrix of telescopes, $H_{bt}\in {\mathbb{R}}^{6\times 4}$ denotes the coupling matrix between satellite body and telescopes.

According to Lagrange’s equation, finally we can obtain the dynamic model of the satellite platform:

(10) $$H\left(q\right)\ddot{q}+C\left(q,\dot{q}\right)\dot{q}=Q$$

where $H\left(q\right)$ denotes the inertia matrix, $C\left(q,\dot{q}\right)$ denotes centrifugal and Coriolis effects, Q denotes generalized force.

Consider disturbance, we have:

(11) $$H\left(q\right)\ddot{q}+C\left(q,\dot{q}\right)\dot{q}=\tau +d$$

where ***d*** is a vector of unknown disturbances.

There are some properties for this equation:

**Property** **1.**  $\dot{H}\left(q\right)$*is symmetric and a positive definite,*$H\left(q\right)=H{\left(q\right)}^{T}$*and*$x^{T}H\left(q\right)x>0$.

**Property** **2.**  $\dot{H}\left(q\right)-2C\left(q,\dot{q}\right)$*is a skew-symmetric matrix, namely*$x^{T}\left(\dot{H}\left(q\right)-2C\left(q,\dot{q}\right)\right)x=0$.

Property 1 is obvious because the kinetic energy $T=\frac{1}{2}{\dot{q}}^{T}H\dot{q}$ is positive for any $\dot{q}\neq 0$ and Property 2 can be proved by the kinetic energy theorem with the identity which is expressed as $\frac{1}{2}\frac{d}{dt}\left[{\dot{q}}^{T}H\dot{q}\right]={\dot{q}}^{T}\tau$.

## 4. Controller Design

### 4.1. Predefined-Time Stable Theory

Consider the nonlinear autonomous dynamical system:

(12) $$\dot{x}=f\left(x\right)$$

where $x\in {\mathbb{R}}^{n}$ denotes the state vector and f: ${\mathbb{R}}^{n}\to {\mathbb{R}}^{n}$ is a nonlinear and locally Lebesgue-integrable function, with $f\left(0\right)=0$. The initial conditions are denoted by $x_{0}=x\left(0\right)\in {\mathbb{R}}^{n}$.

**Definition** **1.**  *(Globally finite-time stable): System (12) is globally finite-time stable if it is globally asymptotically stable and any solution*$x\left(t,x_{0}\right)$*of (12) reaches the equilibrium point at some finite time moment, that is*$\forall x\geq T\left(x_{0}\right)$: $x\left(t,x_{0}\right)=0$*, where*T: ${\mathbb{R}}^{n}\to {\mathbb{R}}_{+}\cup \left\{0\right\}$*is called the settling-time function.*

**Definition** **2.**  *(Fixed-time stable): System (12) is fixed-time stable if it is globally finite-time stable and the settling-time function is bounded,*$\exists T_{\max}>0$: $\forall x_{0}\in {\mathbb{R}}^{n}$: $T\left(x_{0}\right)\leq T_{\max}$.

**Definition** **3.**  *(Predefined-time stable): System (12) is predefined-time stable if it is fixed-time stable and exists a predefined constant* $T_{C}>0$ *, the settling-time function* T *satisfies*

(13) $$T\left(x_{0}\right)\leq T_{C},\forall x_{0}\in {\mathbb{R}}^{n}$$

$T_{C}$ *is called a predefined time.*

The predefined-time stability can be attained by a method similar to the Lyapunov method, which can be summarized as the following theorems:

**Theorem** **1.**  *(Lyapunov function-like method of predefined-time stabilization): Consider the uncertain dynamical system:*

(14) $$\dot{x}=f\left(t,x,d\right)$$

*where* $d\in {\mathbb{R}}^{n}$ *is a vector of unknown disturbances and dynamic uncertainties. If there exists a radially unbounded Lyapunov function* $V\left(x\right)$: ${\mathbb{R}}^{n}\to {\mathbb{R}}_{+}\cup \left\{0\right\}$*, and it satisfies the following properties [39]:*

(1) *V*(***x***) = 0 *if and only if****x*** = 0.
(2) *V*(***x***) ≥ 0 *for any* ***x***.
(3) *For* ∀***x****, the derivative of* *V*(***x***)* satisfies:*

(15) $$\dot{V}\leq -\frac{\pi }{\eta \gamma \beta T_{C}}\left(\gamma^{2}V^{1-\eta /2}+\beta^{2}V^{1+\eta /2}\right)$$

*where* $T_{C}>0$ *is a predefined-time constant and* $\eta \in \left(0,1\right)$, γ>0, β>0 *are parameters. Then, system (14) is globally predefined-time stable with predefined time* $T_{C}$.

**Proof :** 

According to (15), we have:

(16) $$\frac{dV}{dt}\leq -\frac{\pi }{\eta \gamma \beta T_{C}}\left(\gamma^{2}V^{1-\eta /2}+\beta^{2}V^{1+\eta /2}\right)$$

If *V*(***x***) = 0, Equation (15) is obvious. Consider that *V*(***x***) > 0, we have:

(17) $$\frac{\pi }{\eta \gamma \beta T_{C}}\left(\gamma^{2}V^{1-\eta /2}+\beta^{2}V^{1+\eta /2}\right)>0$$

Then Equation (16) will be:

(18) $$\frac{dV}{-\frac{\pi }{\eta \gamma \beta T_{C}}\left(\gamma^{2}V^{1-\eta /2}+\beta^{2}V^{1+\eta /2}\right)}\geq dt$$

By integrating the Equation (21), we can obtain:

(19) $$\begin{array}{cc} T\left(x_{0}\right) & \leq -\frac{\eta \gamma \beta T_{C}}{\pi }\int_{V\left(x_{0}\right)}^{0}\frac{dV}{\left(\gamma^{2}V^{1-\eta /2}+\beta^{2}V^{1+\eta /2}\right)}\\ & =-\frac{\eta \gamma \beta T_{C}}{\pi }\int_{V\left(x_{0}\right)}^{0}\frac{V^{\eta /2-1}dV}{\left(\gamma^{2}+{\left(\beta V^{\eta /2}\right)}^{2}\right)}\\ & =-\frac{\eta \gamma \beta T_{C}}{\pi }\int_{V\left(x_{0}\right)}^{0}\frac{\frac{2}{\eta \beta }d\left(\beta V^{\eta /2}\right)}{\left[\gamma^{2}+{\left(\beta V^{\eta /2}\right)}^{2}\right]}\\ & =-\frac{2T_{C}}{\pi }\arctan\left(\frac{\beta V^{\eta /2}}{\gamma }\right)\left|{}_{V\left(x_{0}\right)}^{0}\right.\\ & =T_{C}\left[\frac{2}{\pi }\arctan\left(\frac{\beta V{\left(x_{0}\right)}^{\eta /2}}{\gamma }\right)\right] \end{array}$$

□

It is obvious that $\lim_{V\left(x_{0}\right)\to \infty }\arctan\left(\frac{\beta V{\left(x_{0}\right)}^{\eta /2}}{\gamma }\right)=\frac{\pi }{2}$ such that $\lim_{V\left(x_{0}\right)\to \infty }T\left(x_{0}\right)=T_{C}$. Therefore, $T_{C}$ is the upper bound of $T\left(x_{0}\right)$, then the origin of system (14) is globally predefined-time stable with predefined-time $T_{C}$.

### 4.2. Controller Design

First, we define an error as:

(20) $$e=q-q_{d}$$

where $q_{d}$ is the desired value of the satellite attitude angle and telescope angle, q is the actual value of the satellite attitude angle and telescope angle.

We assume a reference denoted as ${\dot{q}}_{r}$, the control goal is $\dot{q}\to {\dot{q}}_{r}$. We design the reference as:

(21) $${\dot{q}}_{r}={\dot{q}}_{d}-\alpha e$$

where α>0 is a gain parameter, it is obvious that if $\dot{q}={\dot{q}}_{r}$ this will result in $q\to q_{d}$. Moreover, we assume that $q_{d}$ is second-order differentiable under any conditions.

The extended error can be denoted as:

(22) $$S_{r}=\dot{q}-{\dot{q}}_{r}$$

We have:

(23) $$Y_{r}=H\left(q\right){\ddot{q}}_{r}+C\left(q,\dot{q}\right){\dot{q}}_{r}-d$$

Then the dynamic equation becomes:

(24) $$H\left(q\right){\dot{S}}_{r}+C\left(q,\dot{q}\right)S_{r}=\tau -Y_{r}$$

We choose the Lyapunov function as [37]:

(25) $$V=\frac{1}{2}S_{r}{}^{T}H\left(q\right)S_{r}$$

We design the control torque as:

(26) $$\tau =-\frac{\pi }{2\eta \gamma \beta T_{c}}\left(\gamma^{2}V^{-\eta /2}+\beta^{2}V^{+\eta /2}\right)H\left(q\right)S_{r}+Y_{r}$$

Derivative *V* yields:

(27) $$\begin{array}{cc} \dot{V} & =S_{r}^{T}H\left(q\right){\dot{S}}_{r}+\frac{1}{2}S_{r}^{T}\dot{H}\left(q\right)S_{r}\\ & =S_{r}^{T}\left[\tau -Y_{r}-C\left(q,\dot{q}\right)S_{r}\right]+S_{r}^{T}C\left(q,\dot{q}\right)S_{r}\\ & =S_{r}^{T}\left(\tau -Y_{r}\right)\\ & =S_{r}^{T}\left[-\frac{\pi }{2\eta \gamma \beta T_{c}}\left(\gamma^{2}V^{-\eta /2}+\beta^{2}V^{+\eta /2}\right)H\left(q\right)S_{r}\right]\\ & =-\frac{\pi }{2\eta \gamma \beta T_{c}}\left(\gamma^{2}V^{-\eta /2}+\beta^{2}V^{+\eta /2}\right)S_{r}^{T}H\left(q\right)S_{r}\\ & =-\frac{\pi }{2\eta \gamma \beta T_{c}}\left(\gamma^{2}V^{-\eta /2}+\beta^{2}V^{+\eta /2}\right)\cdot \left(2V\right)\\ & =-\frac{\pi }{\eta \gamma \beta T_{c}}\left(\gamma^{2}V^{1-\eta /2}+\beta^{2}V^{1+\eta /2}\right) \end{array}$$

This indicates that the controller is predefined-time stable.

## 5. Numerical Simulations

To demonstrate the effectiveness of the acquisition scheme and the control law to the satellite platform, we developed a nonlinear simulator with three satellite platforms including ground stations.

### 5.1. Initial Conditions

The simulator consisted of three individual satellites and a ground station and every satellite contained a multibody system nonlinear dynamic model, a noise model and a controller, connected to the ground station via data communication. The nonlinear dynamic model for the platform with 10 DOFs included a satellite and telescopes, while the dynamics of the test masses were neglected which are regarded as initial sensors and provide high-precision attitude information for spacecraft. The interaction between the test masses and satellite was regarded as a disturbance to add to the dynamic model of the platform. The noise of actuators and sensors which was discussed in Section 2.3 was considered in the dynamic model.

**Figure 10:**
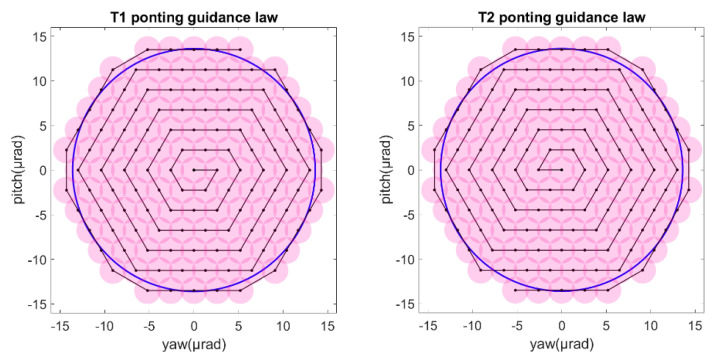
Guidance law for T1 and T2 on satellite A.

We assumed the uncertainty half-cone was 13.6 μrad while the laser beam half-cone was about 1.5 μrad [24]. Then we could obtain the guidance law for the T1 and T2 of satellite A. The detail of the guidance law is show in Figure 10.

We assumed that the waiting time at each point was 6s which was enough for the response of the opposite satellite and the predefined stable time for the controller was 5s. The initial direction of SC for T1 of SA was [5.9 −2.8] × 10^−6^ rad and SB for T2 of SA was [−6.2 7.6] × 10^−6^ rad. The initial direction of SC for T2 of SC was [6 −10.4] × 10^−6^ μrad. The total simulation time was 1000 s, and the step size was 0.1 s.

### 5.2. Simulation Results

The L1/L1′ and L2/L2′ were established at the same time, then L3/L3′ was established.

(1) The establishment of L1/L1′

**Figure 11:**
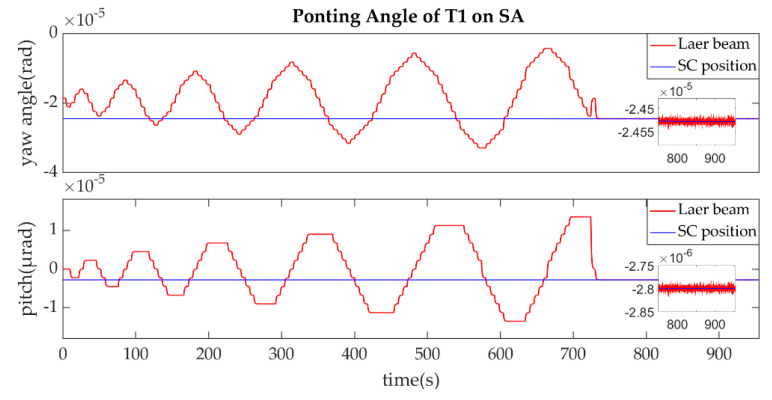
Pointing Angle of T1 on SA.

**Figure 12:**
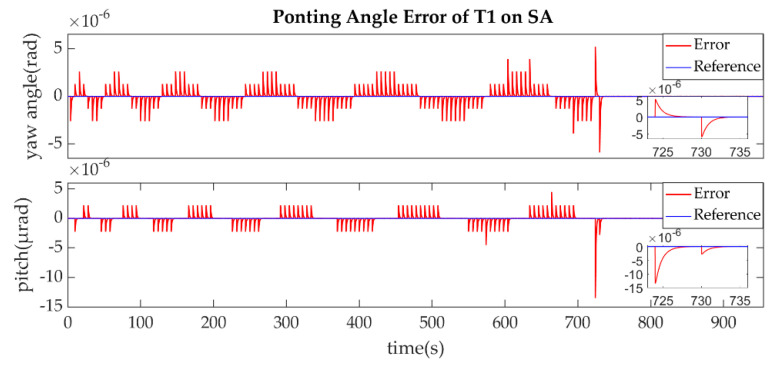
Pointing Angle Error of T1 on SA.

**Figure 13:**
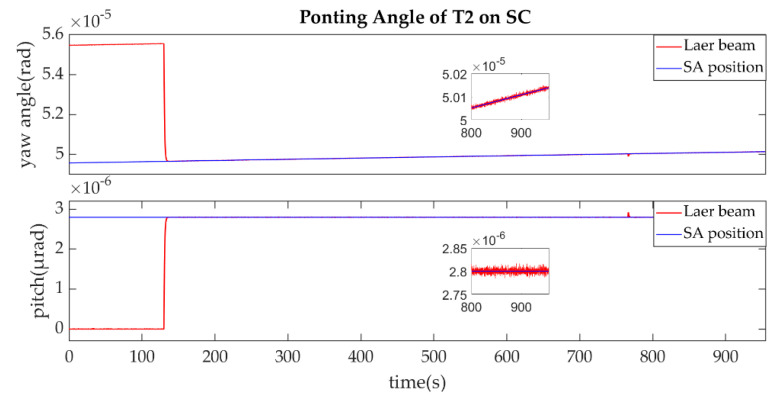
Pointing Angle of T2 on SC.

**Figure 14:**
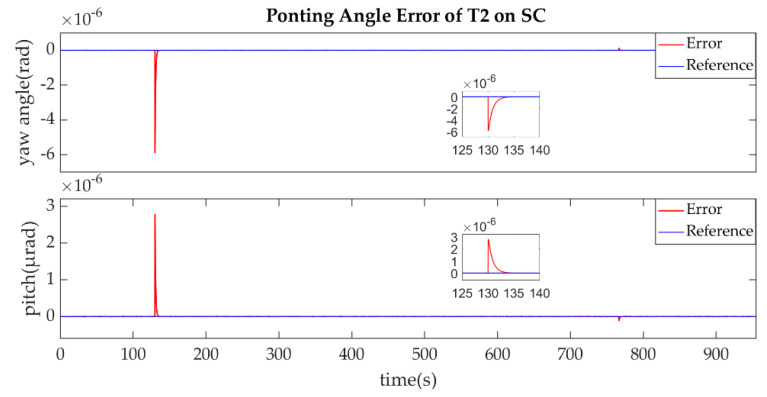
Pointing Angle Error of T2 on SC.

The establishment of L1/L1′ is shown in Figure 11, Figure 12, Figure 13 and Figure 14.

As described in the Figure 3, the ends of L1/L1′ were the T1 of SA and T2 of SC. Figure 11 shows that the T1 of SA was the master end to scan the uncertainty cone of SC with the hexagonal scanning guidance law. Figure 12 shows the angle error of T1 on SA. Figure 13 shows the T2 of SC as the slave end to stare the uncertainty cone of SA and to wait for the laser signal from the T1 of SA. When it received the laser signal, it controlled the direction of its T2 to point the direction of the laser beam and this occurred at about 130 s from Figure 14. When the T1 of SA finished the scanning, it found the laser beam from T2 of SC and controlled its T1 to point SA, then the laser link between SA and SC namely L1/L2′ was stablished at about 735 s. It can be observed from the partial enlargement of Figure 11 and Figure 13 that the precision of the results was better than 0.1 μrad. The partial enlargement of Figure 12 and Figure 14 indicate that the controller could meet the requirements where the settling time was less than 5 s.

(2) The Establishment of L2/L2′

**Figure 15:**
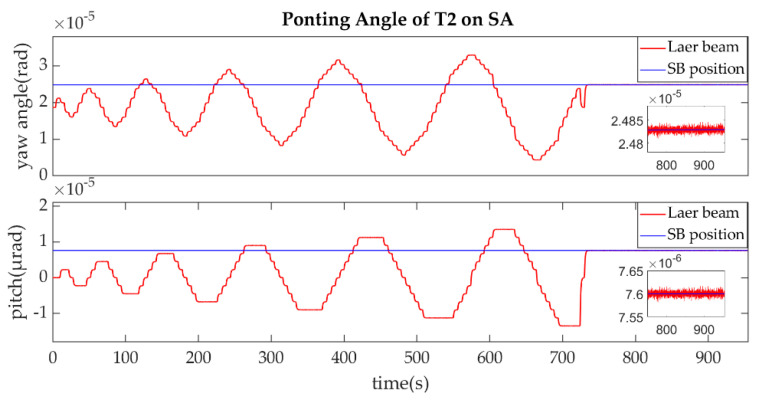
Pointing Angle of T2 on SA.

**Figure 16:**
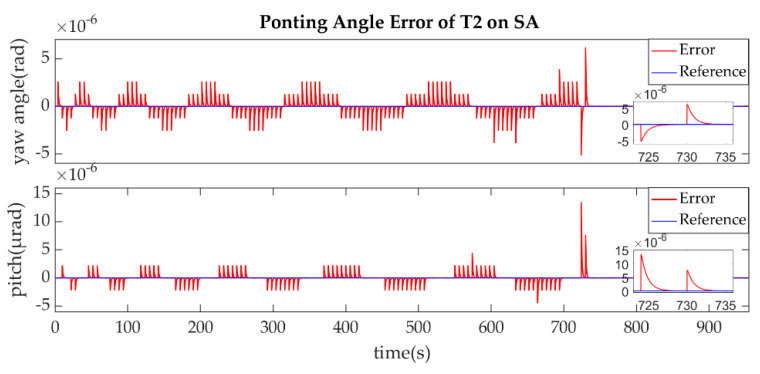
Ponting Angle Error of T2 on SA.

**Figure 17:**
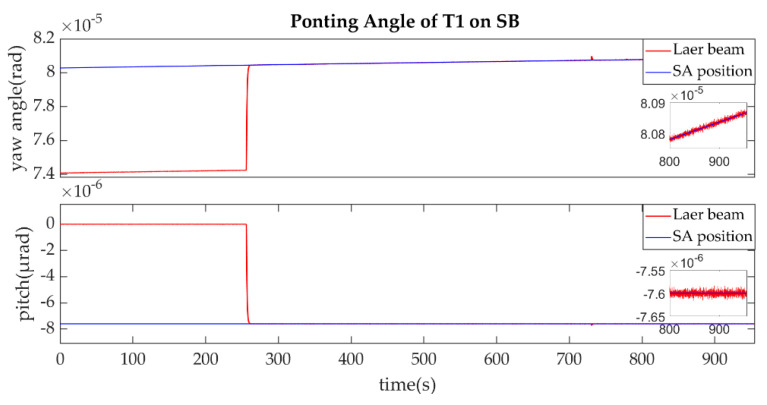
Ponting Angle of T1 on SB.

**Figure 18:**
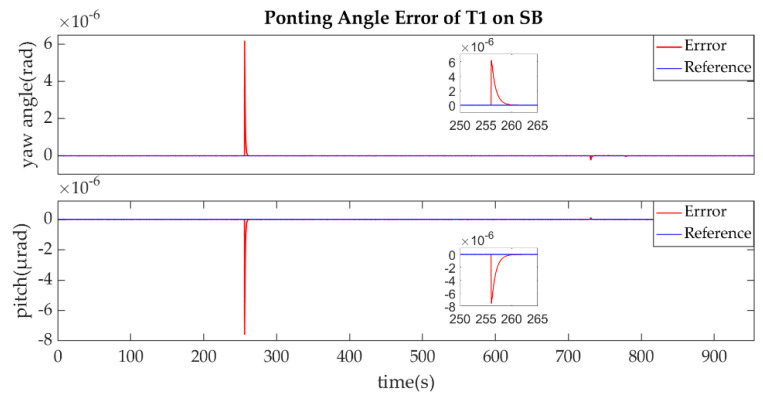
Ponting Angle Error of T1 on SB.

The establishment of L2/L2′ is shown in Figure 15, Figure 16, Figure 17 and Figure 18.

The ends of L2/L2′ are the T2 of SA and T1 of SB, the same as L1/L1′ link. The T2 of SA was the master end to scan the uncertainty cone of SB with the hexagonal scanning guidance law is shown in Figure 15. Figure 16 shows the angle error of T2 on SA. Figure 17 shows the T1 of SB as the slave end to stare the uncertainty cone of SA and to wait for the laser signal from the T2 of SA. When it received the laser signal, it controlled its T1 to point the direction of the laser beam, this occurred at about 256 s from Figure 18. When the T2 of SA finished the scanning, it found the laser beam from T2 of SC and controlled its T2 to point SA, then the laser link between SA and SC namely L2/L2′ was stablished at about 735 s which is same to the L1/L1′. It can be observed from the partial enlargement in Figure 15 and Figure 17 that the precision of the results was better than 0.1μrad and the partial enlargements in Figure 16 and Figure 18 indicate that the controller could meet the requirements where the settling time was less than 5 s.

(3) The Establishment of L3/L3′

**Figure 19:**
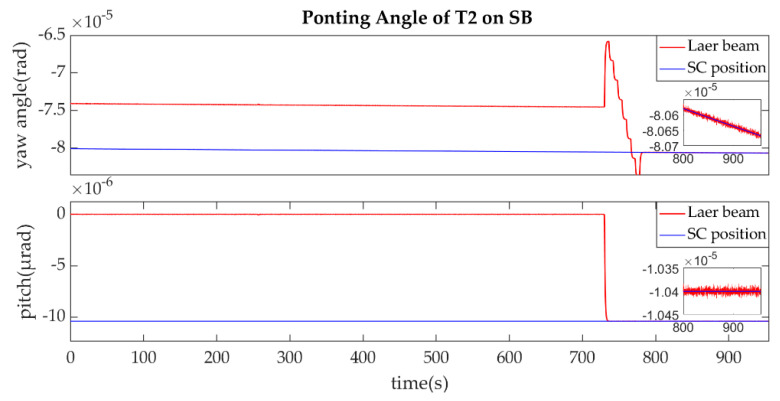
Ponting Angle of T2 on SB.

**Figure 20:**
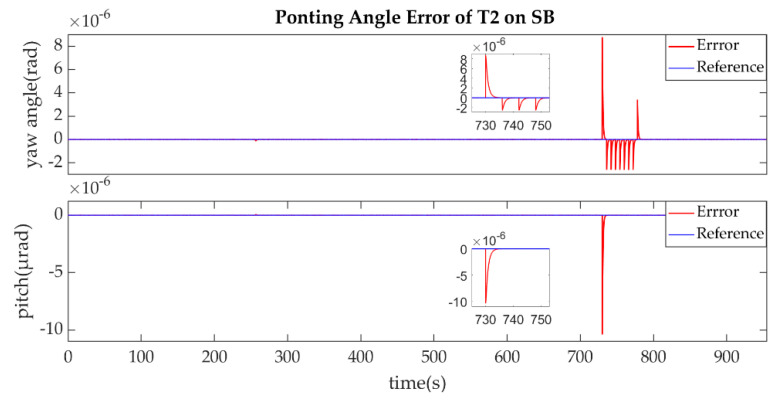
Ponting Angle Error of T2 on SB.

**Figure 21:**
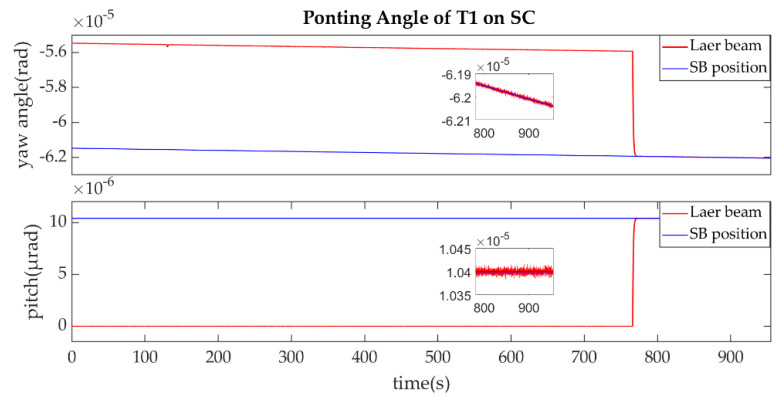
Ponting Angle of T1 on SC.

**Figure 22:**
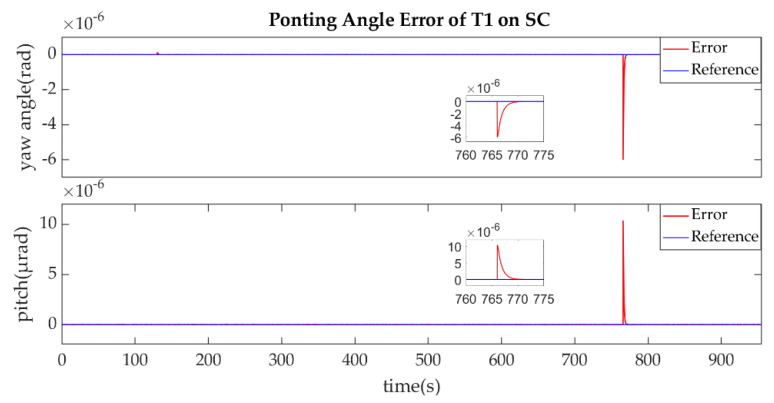
Ponting Angle Error of T1 on SC.

The establishment of L3/L3′ is showed in Figure 19, Figure 20, Figure 21 and Figure 22.

During the establishment of laser link, these three satellites were in constant communication with the ground station, so when SA received the laser signal, it sent the direction information of SB and SC to the ground station. The ground station processed the information and calculated the guidance law to cover the uncertainty cone of SC, then sent it to SB. The purpose of this operation was to reduce the acquisition time. Then SB controlled its T2 and scanned the processed uncertainty cone of SC with the new scanning guidance law while SC stared the uncertainty cone of SA waiting for the laser beam. Figure 19 shows the time series of the pointing angle of T2 on SB. We found that the guidance law was simpler and the acquisition time was shorter. Figure 21 shows the time for the T1 of SC to receive the incoming laser beam was about 765 s. The partial enlargements in Figure 19, Figure 21, Figure 20 and Figure 22 indicate that the precision of the results and controller performance could satisfy the requirements of acquisition.

**Figure 23:**
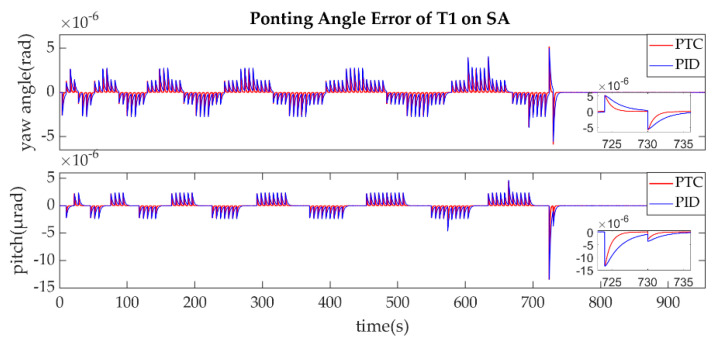
Comparison for the Pointing Angle Error of T1 on SA.

The comparison between the proposed predefined-time controller which is denoted as PTC and the traditional proportional-integral-derivative controller which is denoted as PID is shown in Figure 23.

It can be observed that the PTC controller could guarantee the settling time within 5 s while PID controller could not at some points. This simulation indicates that the PTC controller has an advantage in prescribed-time convergence for the acquisition phase over the PID controller. Another obvious advantage is that the predefine time of PTC controller can be changed as a parameter according to the requirements of the actual scheme.

## 6. Conclusions

In this paper, a new acquisition scheme was proposed to reduce the acquisition time and a predefined-time controller have been designed to guarantee the control performance. A constellation-wide nonlinear simulator including the ground station was developed to verify the acquisition scheme and control strategy. The time domain simulation results verified the time efficiency of the acquisition scheme: the whole process only took about 13 min, much less than the 70 min in [23]. The results also indicated that the predefined-time controller had great advantages in prescribed-time convergence over the PID controller. Furthermore, the settling time of the predefined-time controller can be changed as a parameter according to our practical requirements.

## Figures and Tables

**Table 1:** Main space GW detection programs all over the world.

Orbit Type	Mission Concept	Number of Satellites	Orbit Configuration	Arm Length
Solar Orbit	LISA [5]	3	Earth-like solar orbits with 20° lag	2.5 × 10^6^ km
	ASTROD-GW [8]	3	Near Sun-Earth L3, L4, L5 points	2.6 × 10^8^ km
	DECIGO [9]	12	Earth-like solar orbits	1 × 10^3^ km
	Big Bang Observer [10]	12	Earth-like solar orbits	5 × 10^4^ km
	ALIA [11]	3	Earth-like solar orbits	5 × 10^5^ km
	TAIJI [12]	3	Earth-like solar orbits	3 × 10^6^ km
Earth Orbit	OMEGA [13]	6	6 × 10^5^ km height earth orbit	1 × 10^6^ km
	gLISA/GEOGRAWI [14]	3	Geostationary orbit	7.3 × 10^4^ km
	GADFLI [15]	3	Geostationary orbit	7.3 × 10^4^ km
	LAGRANGE [16]	3	Earth-Moon L3, L4, L5 points	6.6 × 10^4^ km
	TIANQIN [6]	3	1 × 10^5^ km height earth orbit	1.7 × 10^5^ km

**Table 2:** The relationship among LDA, UCA and FOV for the four acquisition modes.

Stare/Stare	Stare/Scan	Scan/Stare	Scan/Scan
LDA > UCA	LDA < UCA	LDA > UCA	LDA < UCA
UCA < FOV	UCA < FOV	UCA > FOV	UCA > FOV

## Data Availability

Not applicable.
